# Effects of Ginkgo diterpene lactone gluconate injection combined with vascular recanalization on patients with large-vessel occlusive cerebral infarction: A clinical observation study

**DOI:** 10.1097/MD.0000000000045091

**Published:** 2025-10-17

**Authors:** Zhaohai Feng, Meiying Li, Zhide Li, Liangxu Xiang, Jing Xu, Jiachuan Wang

**Affiliations:** a Department of Neurology, Ma’anshan People’s Hospital, Anhui Province, China; b Department of Pathology, Shenzhen Traditional Chinese Medicine Hospital, The Fourth Clinical Medical College of Guangzhou University of Chinese Medicine, Shenzhen, China.

**Keywords:** endovascular recanalization therapy, Ginkgo diterpene lactone glucoside, large-vessel occlusive cerebral infarction, malignant cerebral edema

## Abstract

This study aims to evaluate the clinical efficacy and safety of combining Ginkgo diterpene lactone glucoside injection with endovascular recanalization therapy in patients with large-vessel occlusive cerebral infarction. A total of 80 patients with large-vessel occlusive cerebral infarction who underwent endovascular treatment in the Department of Neurology from January 2021 to December 2022 were selected for this study. The patients were randomly divided into an observation group (n = 40) and a control group (n = 40). The observation group received endovascular recanalization therapy combined with Ginkgo diterpene lactone glucoside injection for 7 days, while the control group received endovascular recanalization therapy without the addition of Ginkgo diterpene lactone glucoside injection. This study assessed the prognosis of both groups after 90 days of treatment. Before treatment, there was no statistically significant difference between the 2 groups in baseline characteristics, such as neurological function (National Institutes of Health Stroke Scale score). However, after 90 days of treatment, there was a significant difference in National Institutes of Health Stroke Scale scores between the observation group and the control group (4.94 ± 3.65 vs 7.74 ± 6.46, *P* = .033). Although there was no statistically significant difference in the proportion of patients with good prognosis (Modified Rankin Scale (mRS) score 0–2) after 90 days of treatment (*P* = .072), the proportion of patients with good prognosis in the observation group was higher than that in the control group (65% vs 45%). After 90 days of treatment, there was also no statistically significant difference in the proportion of patients with poor prognosis (mRS score 5–6, where 6 points indicate death) (*P* = .084). However, the proportion of patients with poor prognosis in the observation group was lower than that in the control group (20% vs 37.5%). The comparisons of symptomatic intracranial hemorrhage, malignant cerebral edema, and mortality rates between the 2 groups after treatment did not show statistically significant differences (all *P* > .05). For patients with large-vessel occlusive cerebral infarction who have undergone endovascular recanalization therapy, the combined use of Ginkgo diterpene lactone glucoside injection can effectively improve neurological function and may enhance quality of life without increasing the risk of symptomatic intracranial hemorrhage. No statistically significant reduction in mortality was observed.

## 1. Introduction

Stroke has become the leading cause of death in China, with high mortality and disability rates.^[1]^ The incidence of stroke in China is increasing annually, and there is a trend toward younger patients. Specifically, the proportion of those under 70 years old is on the rise. Among new stroke cases, ischemic stroke accounts for about 70% to 80%, making the quality of treatment for acute ischemic stroke crucial to the health of Chinese residents.^[2]^ In particular, large-vessel occlusive ischemic stroke has extremely high mortality and disability rates. Currently, intravenous recombinant tissue plasminogen activator (rt-PA) thrombolysis and endovascular treatments for large-vessel occlusive ischemic stroke, such as mechanical thrombectomy combined with intravenous thrombolysis bridging or direct mechanical thrombectomy, are considered effective measures to reduce mortality and disability levels.^[3,4]^ However, even with early revascularization, many patients with large-vessel occlusive ischemic stroke still have poor outcomes. To improve patient outcomes after revascularization, it is necessary to enhance endovascular techniques and provide comprehensive drug therapy, including neuroprotective agents and free radical scavenging agents, during the later stages.

Traditional Chinese medicines, such as Ginkgo biloba preparations, have been widely used in ischemic cerebrovascular diseases.^[5]^ Their possible mechanism involves reducing cerebral ischemia-reperfusion injury and improving neurological deficits through inhibition of platelet-activating factor (PAF) activity and neuroprotective effects.^[6]^ The active ingredients of Ginkgo Diterpene Lactone Meglumine Injection are extracted from ginkgo leaves and mainly consist of 3 terpene lactone compounds: ginkgolide A (GA), ginkgolide B (GB), and ginkgolide K (GK). These compounds collectively inhibit the PAF pathway, which is a key antithrombotic and anti-inflammatory mechanism, and regulate neurotrophic factors, such as IGFBP2, to promote repair.^[7]^ For example, in the middle cerebral artery occlusion model, ginkgolide B has been shown to reduce the infarct area and improve behavioral scores.^[8]^ Studies have also demonstrated that combining Ginkgo Diterpene Lactone Meglumine Injection with recombinant tissue plasminogen activator (rt-PA) thrombolysis can increase the clinical effectiveness rate and reduce neurological deficits.^[5]^ But there are currently no reports on the efficacy of traditional Chinese medicine preparations in patients after vascular recanalization. This study aims to evaluate the clinical efficacy and safety of Ginkgo diterpene lactone glucamine injection in combination with vascular recanalization therapy (including mechanical thrombectomy combined with intravenous thrombolysis bridging or direct mechanical thrombectomy) for patients with large-vessel occlusive stroke.

## 2. Subjects and methods

### 2.1. Subjects

From January 2021 to August 2022, patients with acute large-artery occlusive cerebral infarction admitted to the Department of Neurology, Ma’anshan People’s Hospital were enrolled. They met the diagnostic criteria for cerebral infarction and were confirmed by head Computed Tomography to have no bleeding or space-occupying lesions.

### 2.2. Inclusion criteria

Patients diagnosed with acute cerebral infarction within 24 hours of symptom onset; Symptoms persist from disease onset until initiation of revascularization therapy; National Institutes of Health Stroke Scale (NIHSS) score ≥ 6 before treatment; Emergency cerebrovascular examination (cranial Computed Tomography Angiography or Digital Subtraction Angiograph) shows occlusion of major arteries (including the internal carotid artery, middle cerebral artery M1 segment, and vertebrobasilar artery), and the occluded vessel is the responsible vessel for the cerebral infarction; Stroke occurs within 24 hours of symptom onset or is discovered after waking within 24 hours; for strokes occurring between 6 and 24 hours after symptom onset or upon waking, multimodal imaging assessment is required, and patients must meet the DAWN or DEFUSE-3 endovascular treatment criteria^[9,10]^; mRS score before stroke is 0 to 1; Age range 18 to 85 years old; If other diseases are present, the patient’s expected survival time is more than 6 months; Written informed consent was obtained from the patient’s family. The study complies with the standards set by the Ethics Committee of the Ma’anshan People’s Hospital (People’s Hospital of Ma’anshan City Medical Ethics (2020) No. 012-042).

### 2.3. Exclusion criteria

Patients with unstable vital signs after vascular recanalization; patients with severe heart, liver, or kidney diseases; patients at risk of death at any time; patients with a history of cerebral hemorrhage (including subarachnoid hemorrhage); patients with a high risk of bleeding after vascular recanalization treatment; and patients with other contraindications to intravenous thrombolysis and endovascular treatment.

### 2.4. Treatment and grouping

Patients with acute large-vessel occlusive cerebral infarction were treated with revascularization therapy, including intravenous thrombolysis, mechanical thrombectomy, or direct mechanical thrombectomy. Those who achieved TIMI flow grade 2b or 3 were then randomly assigned, using a random number grouping method, to the observation group (Ginkgo diterpene lactone group) or the control group (non-Ginkgo diterpene lactone group). For patients with an onset time of 0 to 4.5 hours, intravenous thrombolysis bridging to mechanical thrombectomy was administered for revascularization. For patients with an onset time of 4.5 to 24 hours or with post-awakening stroke, mechanical thrombectomy was used for revascularization. After revascularization, all patients received Butylphthalide in Sodium Chloride Injection and Statins. For patients receiving bridging therapy, a cranial Computed Tomography scan was performed 24 hours after intravenous thrombolysis. If no bleeding was found, antithrombotic or anticoagulant therapy (for cardioembolic stroke) was initiated. For non-bridge-treated patients, if intracranial hemorrhage was ruled out by imaging after revascularization, antithrombotic or anticoagulant therapy was immediately initiated. Patients who underwent emergency stent treatment were continuously administered ticlopidine for 24 hours. All patients managed their risk factors, including blood pressure, blood glucose, and other comorbidities. The observation group received Ginkgo diterpene lactone glucamine injection starting within 2 hours after thrombectomy, whereas the control group did not receive Ginkgo diterpene lactone glucamine injection. Ginkgo diterpene lactone meglumine injection, 5 mL (containing 25 mg ginkgo diterpene lactone) per dose, should be taken once a day for 7 days. The drug is provided by Jiangsu Kangrong Pharmaceutical Co., Ltd. Before use, slowly add the drug to 250 mL of 0.9% sodium chloride solution to dilute. Then, infuse the solution slowly intravenously.

### 2.4. Outcomes

The primary endpoint was a good outcome, defined as an mRS score of 0–2 or an NIHSS score improvement >4 points at 90 days after treatment. The secondary endpoints included all-cause mortality within 90 days, symptomatic intracerebral hemorrhage, and malignant cerebral edema (midline shift ≥ 5 mm within 72 hours after endovascular treatment).

### 2.5. Statistical analysis

Statistical analysis was performed using SPSS 27.0 software (Chicago). Measurement data were expressed as mean ± standard deviation. The *t*-test was used for inter-group comparisons, and the χ² test or Fisher exact test was used for comparison of categorical data.

## 3. Results

### 3.1. Baseline characteristics

**Figure 1.**
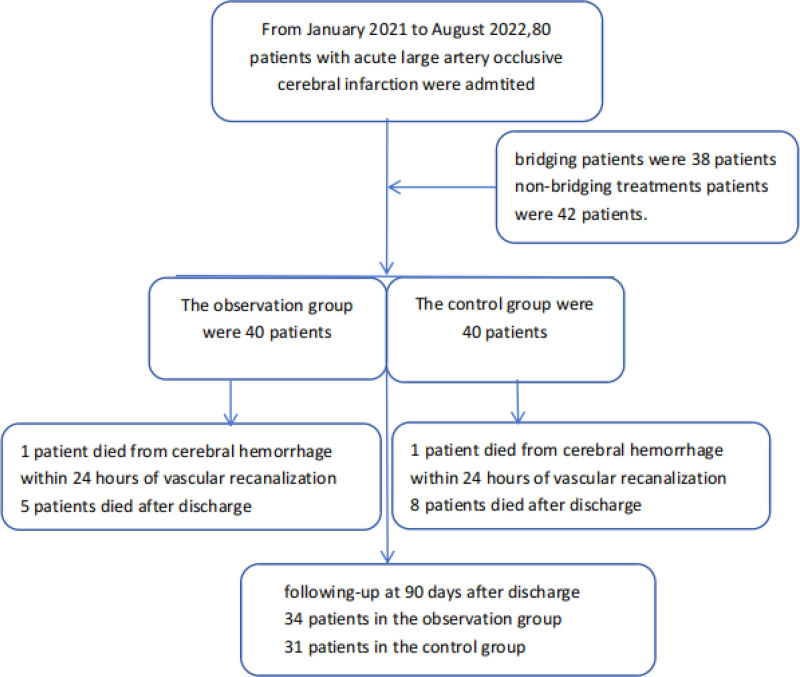
Flow diagram of participants in the study.

A total of 80 patients were enrolled, with 40 and 40 patients in the observation and control groups, respectively. The total number of bridging patients (including 8 patients transferred from primary stroke centers after thrombolysis) was 38. Meanwhile, 42 patients received non-bridging treatments (Fig. 1). The baseline characteristics of the enrolled patients are summarized in Table 1. There were no significant differences between the observation and control groups regarding gender, age, hypertension, atrial fibrillation, diabetes, and dyslipidemia. Furthermore, before treatment, there were no significant differences between the 2 groups in NIHSS and mRS scores. After revascularization, no significant differences were observed in TIMI flow grades. Additionally, there were no significant differences in the proportion of different vascular occlusions or in the methods of revascularization (bridging or non-bridging treatments).

**Table 1:** Comparison of baseline clinical data between the 2 groups.

		Observation group	Control group	t/χ²	*P* value
Gender, man/female (n/n)		26/14	20/20	0.808	.369
Age, years		69.35 ± 10.00	66.55 ± 11.58	1.151	.253
Hypertension, n (%)		24 (60%)	18 (45%)	1.805	.179
Diabetes, n (%)		18 (45%)	14 (35%)	0.833	.361
Abnormal blood lipid, n (%)		10 (25%)	6 (15%)	1.250	.264
AF, n (%)		26 (65%)	21 (50.2%)	1.289	.256
NIHSS score		16.77 ± 5.77	16.92 ± 5.61	-0.118	.907
locked blood vessel	ICA, n (%)	14 (45%)	8 (20%)	2.258	.133
	MCA, n (%)	20 (50%)	28 (70%)	3.333	.068
	VA-BA, n (%)	6 (15%)	4 (10%)	0.457	.499
Number of bridges, n (%)		22 (55%)	16 (40%)	1.805	.179
Grade 3 blood flow, n (%)		36 (90%)	38 (95%)	0.729	.396

AF = atrial fibrillation, ICA = internal carotid artery, MCA = middle cerebral artery, NIHSS = National Institutes of Health Stroke Scale, VA-BA = vertebrobasilar artery.

### 3.2. Main and secondary endpoint events after treatment

**Figure 2.**
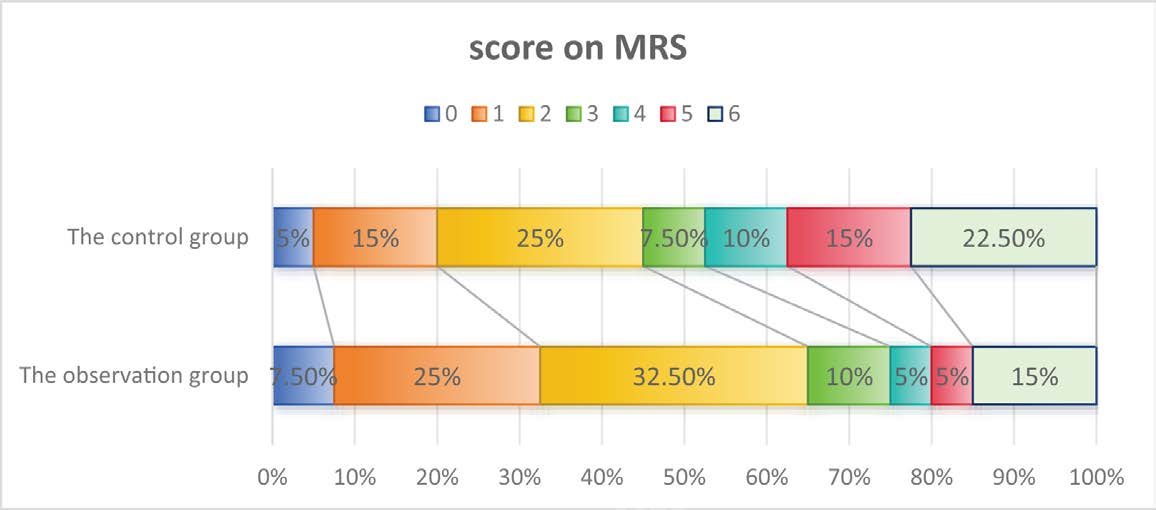
Distribution of mRS scores between observation and control groups at 90 days after treatment.

In both the observation and the control groups, 1 patient experienced symptomatic bleeding within 24 hours of vascular recanalization and was discharged (including deaths) (see Table 2). Within 90 days, 6 patients in the observation group and 9 patients in the control group died. Thus, at 90 days, 34 patients in the observation group and 31 patients in the control group had completed the follow-up. There was a statistically significant difference in NIHSS scores between the 2 groups after 90 days (*P* = .033). Good prognosis: Patients with a mRS score of 0 to 2 were 26 (65%) in the observation group and 18 (45%) in the control group. Although there was no statistical difference (*P* = .072), the proportion of patients with an mRS score of 5 to 6 (6 points for death) was higher in the observation group (Fig. 2). The proportion of patients with an mRS score of 5 to 6 (6 points for death) in the observation group was 8, compared to 15 in the control group, with no statistically significant difference (*P* = .084), but the proportion in the observation group was significantly lower than in the control group (20% vs 37.5%) (Fig. 2). There was no statistically significant difference between the 2 groups in patients with an improvement in NIHSS score >4 points (including those with an mRS score of 0–2) (*P* = .084). There was no statistically significant difference between the 2 groups in the incidence of malignant brain edema, symptomatic intracerebral hemorrhage, and mortality (*P* values: .288, .644, .390, respectively).

**Table 2:** Neurological outcomes and complications at 90 days after treatment in the 2 groups.

		Observation group	Control group	*t*/ χ²	*P*-value
NIHSS score		4.94 ± 3.65	7.74 ± 6.46	−2.17	.033*
mRS score	0–2	26 (65%)	18 (45%)	2.232	.072
	5–6	8 (20%)	15 (37.5%)	2.990	.084
NIHSS score improved (≥ 4 points), n (%)		32 (80%)	25 (60.2%)	2.990	.084
sICH, n (%)		2 (5%)	3 (7.5%)	0.213	.644
die, n (%)		6 (15%)	9 (22.5%)	0.738	.390
MBE, n (%)		3 (7.5%)	6 (15%)	1.127	.288

MBE = malignant brain edema, mRS = modified Rankin Scale, NIHSS = National Institutes of Health Stroke Scale, sICH = symptomatic intracranial hemorrhage.

* *P* < .05.

## 4. Discussion

Ischemic stroke is the most common type of stroke and results in local brain tissue hypoxia, which triggers a series of harmful reactions. These reactions include neuronal apoptosis and other forms of cell death, autophagy, inflammatory responses mediated by various inflammatory factors, and brain edema caused by disruption of the blood–brain barrier. All these processes exhibit a cascade effect. Early revascularization, combined with other comprehensive interventions, can help reduce the damage caused by hypoxia. Intravenous thrombolysis (rt-PA) and endovascular mechanical thrombectomy are currently the most effective methods for revascularization. Additionally, antiplatelet drugs, statins, blood pressure, blood sugar control, and neuroprotective treatments are part of the comprehensive treatment plan. Early revascularization aims to restore blood supply as quickly as possible, minimize the infarcted area, and reduce harmful pathological processes such as inflammation and edema after infarction, thereby improving the patient’s clinical outcome and enabling reintegration into society.

However, intravenous thrombolysis is less effective in treating large-vessel occlusive cerebral infarction, with a lower rate of vascular recanalization. Therefore, most patients with large-vessel occlusive cerebral infarctions require combined endovascular recanalization therapy. For patients who have exceeded the time window, endovascular treatment may be considered after multimodal imaging assessment. According to the ANGEL-ACT study (a study conducted in our country), patients with anterior circulation large-vessel occlusive cerebral infarction showed improved neurological function within 90 days after thrombolysis. However, the rate of favorable outcome was only 45%.^[11]^ In other randomized controlled trials of endovascular recanalization therapy, such as the MR CLEAN trial, only 33% of patients had a modified Rankin Scale (mRS) score of 0 to 2 within 90 days. In the HERMES study, which evaluated reperfusion efficacy in multiple endovascular stroke treatments, the overall proportion of patients with an mRS score of 0 to 2 was only 46%.^[4]^ Overall, while endovascular recanalization therapy has improved the prognosis for some patients with large-vessel occlusive cerebral infarction, clinical neurological improvement remains limited, necessitating the use of additional treatment methods to achieve better outcomes.

Traditional Chinese medicine and its preparations have shown broad application potential in the treatment of various diseases, particularly in the early stages of acute stroke. Ginkgo biloba flavonoids and Ginkgo leaf extract are known to possess PAF receptor antagonists (PAFR) properties. These antagonists not only inhibit platelets but also exhibit multiple effects, including anti-inflammatory and neuroprotective benefits.^[12]^ A study suggested that ginkgolide might effectively reduce the recurrence risk of large-vessel atherosclerotic cerebral infarction.^[13]^ Another study noted that in patients with acute cerebral infarction who receive intravenous thrombolysis, combining ginkgolide can improve neurological function without increasing the risk of bleeding.^[14]^ A meta-analysis indicated that Ginkgo biloba flavonoid glucamine injection significantly improves the treatment of acute cerebral infarction and enhances the clinical outcomes of rt-PA intravenous thrombolysis.^[5]^ Further research revealed that ginkgolide protects against brain ischemia-reperfusion injury in rats by reducing neuronal apoptosis and through its anti-inflammatory and anti-oxidative stress properties, thereby improving ischemic nerve damage.^[15]^ The main components of Ginkgo biloba flavonoid glucamine injection include ginkgolides (GA, GB, GK). Their combined use enhances antiplatelet aggregation activity and reduces thrombosis and inflammation, thus improving blood supply to ischemic areas, promoting neural repair in damaged regions, and ultimately enhancing and restoring the neurological function of patients with cerebral infarction.^[16,17]^ The results of many clinical studies show that Ginkgo biloba can significantly improve the clinical outcomes of patients with acute cerebral infarction.^[18]^

Previous studies that used the ginkgo diterpene lactone meglumine injection to treat ischemic stroke have all started treatment 24 hours or 48 hours after the stroke event, with a duration of 14 days.^[5]^ In this study, the medication was administered within 2 hours after reperfusion of the occluded large vessel, earlier than the administration time reported in previous studies. This timing is critical because the first 2 hours after vessel reperfusion represent the peak period of ischemic reperfusion injury. During this period, a large amount of free radicals is generated, intracellular calcium overload occurs, and the inflammatory cascade reaction reaches its peak.^[19]^ Early after reperfusion, swelling of microvascular endothelial cells and platelet activation lead to the no-reflow phenomenon, while the integrity of the blood-brain barrier rapidly deteriorates within this 2-hour window.^[20]^ Ginkgo diterpene lactone, mainly containing GK and GB, can rapidly eliminate oxygen free radicals, inhibit pro-inflammatory factor release, and interrupt the inflammatory cascade. Its active ingredients, such as bilobalide, significantly inhibit platelet aggregation by blocking PAF receptors and improve microcirculation. Additionally, the GB component reduces MMP-9 expression, enhances tight junction protein stability, and lowers the risk of vascular-induced edema.^[19]^

This study shows that after undergoing vascular recanalization therapy, 55% of patients achieved good neurological outcomes (mRS score 0–2) within 90 days, a significantly higher rate compared to previous studies. Patients treated with Ginkgo biloba lactone and glucosamine injection had a 65% rate of good prognosis compared to 45% in the control group. Although there was no statistically significant difference between the 2 groups, the observation group showed a trend toward better outcomes. Before treatment, there was no significant difference in the NIHSS scores between the 2 groups. However, 90 days posttreatment, the NIHSS scores of the observation group were significantly lower than those of the control group (*P *= .032), indicating that the combined use of Ginkgo biloba lactone and glucosamine injection can improve neurological function. Although there was no statistically significant difference in the incidence of cerebral edema between the 2 groups, the incidence of cerebral edema in the observation group was lower than that in the control group, which is considered a critical factor leading to poor outcomes or even death. The incidence of adverse outcomes in the observation group was lower than that in the control group (20% vs 37.5%). These improvements may be attributed to the pharmacological effects of Ginkgo biloba lactone. As a traditional Chinese medicine preparation, Ginkgo biloba lactone and glucosamine injections primarily function to promote blood circulation and improve microcirculation. Studies have shown that Ginkgo biloba lactone can antagonize PAF-induced platelet aggregation and has neuroprotective effects.^[21]^ A study found that early application of Ginkgo biloba in acute cerebral infarction patients significantly improved prognosis; moreover, nepetin has been shown to increase the proportion of patients with a good prognosis after 90 days.^[22]^ Additionally, researchers have noted that Ginkgo biloba lactone and glucosamine injection can improve the quality of life in acute cerebral infarction patients at both 14 and 90 days.^[22,23]^ Furthermore, studies have shown that Ginkgo biloba lactone and glucosamine injection is highly effective in combating cerebral ischemia, possibly by reducing the permeability of the blood-brain barrier to improve cerebral edema.^[24,25]^ Some researchers suggest that Ginkgo biloba lactone and glucosamine injection can reduce the incidence of acute neurological deterioration in cerebral infarction patients.^[26]^ In cases of large-vessel occlusive cerebral infarction, the disruption of the blood-brain barrier is a significant issue, and intravascular treatment may increase its permeability. Therefore, combining Ginkgo diterpene lactone glucamine injections with other treatments may help improve the prognosis for these patients.

In this study, there was no significant difference in the proportion of symptomatic intracranial hemorrhage between the observation group and the control group (*P *= .644), nor was there a significant difference in mortality (*P *= .390). A meta-analysis of 28 randomized controlled studies found that 5 of these studies involved the combined use of rt-PA. This combined treatment significantly improved neurological function after intravenous thrombolysis for acute cerebral infarction without increasing adverse drug reactions or severe complications. Therefore, it is suggested that Ginkgo biloba lactone injection can be used as an adjunctive treatment for intravenous thrombolysis.^[5]^ The results of this study are consistent with these findings regarding the efficacy and safety of adjunctive treatments in acute cerebral infarction.

Based on the analysis, the study’s results indicate that for patients with large-vessel occlusive cerebral infarction, the combination of Ginkgo diterpene lactone glucamine injection after vascular recanalization can improve neurological function at 90 days, enhance quality of life, and reduce mortality without increasing the risk of symptomatic intracranial hemorrhage. However, as this study is a single-center, small-sample study, further large-scale randomized controlled trials are needed to confirm the efficacy and safety of Ginkgo biloba injection combined with vascular recanalization in the treatment of large-vessel occlusive cerebral infarction. It is also necessary to further investigate the impact of ginkgo diterpene lactones combined with different vascular recanalization methods on prognosis.

## Acknowledgments

We thank all participants in this study.

## Author contributions

**Conceptualization:** Zhaohai Feng, Meiying Li.

**Data curation:** Zhaohai Feng, Zhide Li, Liangxu Xiang, Jing Xu.

**Formal analysis:** Meiying Li.

**Funding acquisition:** Jiachuan Wang.

**Investigation:** Zhide Li, Liangxu Xiang, Jing Xu.

**Methodology:** Zhaohai Feng, Jiachuan Wang.

**Writing – original draft:** Zhaohai Feng.

**Writing – review & editing:** Zhaohai Feng, Meiying Li, Jiachuan Wang.
